# Effect of lidocaine perioperative infusion on chronic postsurgical pain in patients undergoing thoracoscopic radical pneumonectomy

**DOI:** 10.1186/s12871-022-01795-2

**Published:** 2022-08-09

**Authors:** Yi Lu, Hehe Ding, Caiqun Shao, Ning Wang, Junhua Shi, Chaohui Lian, Junzheng Wu, Wangning Shangguan

**Affiliations:** 1 Department of Anesthesiology and Perioperative Medicine, The Second Affiliated Hospital and Yuying Children’s Hospital of Wenzhou Medical University, 109 West Xueyuan Road, Wenzhou, 325027 China; 2 Department of Anesthesiology, People’s Hospital of Ruian, The Third Affiliated Hospital of Wenzhou Medical University, Ruian, 325200 Zhejiang, People’s Republic of China; 3 Department of Anesthesia and Pediatrics, Cincinnati Children Hospital Medical Center, Cincinnati, OH USA

**Keywords:** Chronic postoperative pain, Intravenous infusion, Lidocaine, Thoracoscope, Interleukin

## Abstract

**Background:**

Thoracoscopic radical pneumonectomy is associated with a high incidence of postoperative chronic pain. Studies on the benefits of lidocaine intravenous infusion during the perioperative period were still controversial in thoracoscopic surgery.

**Methods:**

Sixty-four lung cancer patients scheduled for thoracoscopic radical pneumonectomy were randomly divided into two groups: normal saline group (control group) or lidocaine group. In the lidocaine group, 1.5 mg/kg lidocaine was administered during the anesthesia induction, and 2 mg·kg^−1^·h^−1^ lidocaine was continuously intravenous infused until the end of the surgery. After the surgery, a mixture of 2 μg/kg sufentanil and 10 mg/kg lidocaine was continuously intravenous infused by postoperative patient-controlled intravenous analgesia pump (100 ml). In the control group, the same volume of normal saline was administered according to the calculation of lidocaine during anesthesia induction, maintenance and postoperative patient-controlled intravenous analgesia. The primary outcome was the incidence of chronic postoperative pain at 3 months after the surgery. The secondary outcomes include the incidence of chronic postoperative pain at 6 months after the surgery; the effect of lidocaine on postoperative pain within the first 24 and 48 h; total amount of sufentanil administered during entire procedure and the number of PCA triggers within 48 h after surgery.

**Results:**

Compared with the control group, the incidence of chronic pain at 3 months after the surgery was significantly lower (13 cases, 46.4% vs. 6 cases, 20.7%, *p* < 0.05), but no significant difference at 6 months between two group. The cumulative dosage of sufentanil in perioperative period was significantly lower (149.64 ± 18.20 μg vs. 139.47 ± 16.75 μg) (*p* < 0.05), and the number of PCA triggers (8.21 ± 4.37 *vs.* 5.83 ± 4.12, *p* < 0.05) was significantly greater in the control group. The NRS pain scores at 24 h (1.68 ± 0.72 vs. 1.90 ± 0.86) and 48 h (1.21 ± 0.42 vs. 1.20 ± 0.41) after the operation were no significant difference.

**Conclusion:**

Perioperative infusion lidocaine significantly reduced the number of PCA triggers and the incidence of chronic postoperative pain at 3 months after the thoracoscopic radical pneumonectomy.

**Trial registration:**

http://www.chictr.org.cn: ChiCTR1900024759, frist registration date 26/07/2019.

## Background

The International Classification of Diseases (ICD-11) definition is that Chronic postsurgical pain (CPSP) is the development or enhancement of pain in the operative area after the surgery, which lasts longer than the healing process (at least 3 months), and cannot be better explained by other reasons such as infection, malignancy or pre-existing pain [1]. CPSP reduces the quality of life of patients after surgery, increases the risk of depression, sleep disorders, and increases the use of anxiety drugs [2]. This pain is not static, it can worsen or subside over time, and it may last for many years after operation [3]. Demographic factors, psychosocial factors, genetics, type of surgery, acute postoperative pain and perioperative analgesia are all related to the occurrence of CPSP [4]. Thoracic surgery, including thoracotomy (one in two patients suffers from CPSP [5]) and thoracoscopic surgery, usually leads to CPSP [6]. Compared with thoracotomy, thoracoscopic surgery can theoretically reduce the incidence of CPSP, but the incidence of CPSP still reaches 20 to 47% [7, 8].

Lidocaine, a short-acting local anesthetic, has been proved to have analgesic and anti-inflammatory effects [9]. The application of lidocaine by continuous infusion in the intraoperative period and immediately after the surgery appears to reduce the immediate postoperative pain, and to prevent the CPSP [10]. Studies have showed that intravenous infusion of lidocaine during abdominal surgery can not only relieve pain, but also reduce the consumption of opioids [11, 12]. The beneficial effects may be related to its ability to suppress inflammatory response [13].

However, studies on the benefits of lidocaine intravenous infusion during the perioperative period were still controversial in thoracoscopic surgery. The purpose of the present study was to determine the effect of perioperative intravenous infusion of lidocaine on CPSP after thoracoscopic radical resection of lung cancer. It was hypothesized that perioperative intravenous infusion of lidocaine could reduce the incidence of CPSP.

## Materials and methods

This prospective, randomized, double-blinded study was approved by the Medical Ethics Committee of the Second Affiliated Hospital and Yuying Children's Hospital of Wenzhou Medical University (No. LCKY2019-07). This study is in accordance with the CONSORT 2010 statement [14]. We would go to the ward the day before the operation and give informed consent to the patient in a separate room. After obtained the written informed consent, 64 patients, ASA II or III, aged 18 to 65 years old, scheduled for elective thoracoscopic radical pneumonectomy were enrolled, and randomly divided into two groups using a computer-generated digit-number program (SAS PLAN; SAS Institute Inc.): normal saline group (control group) and lidocaine group, 32 patients in each group. The exclusion criteria included mental disorders or no cooperation; serious respiratory or cardiovascular disease; local anesthetic drug allergy; a previous history of chest surgery; experiencing hyperalgesia or refractory cancer pain; used of analgesics within 3 months; acute or chronic pain of any cause; planning a second operation or the conversion to thoracotomy.

This study was a double-blind design. The sequential numbers from 1 to 64 for participants were marked outside of each individual envelopes. Based on a computer-generated sequence, 32 paper slips marked with #1 [normal saline group (control group)] and 32 slips marked with #2 (lidocaine group) were sealed inside of envelopes. On the day of study, an investigator who was not involved in the administration and observation was opened an envelope with the smallest sequential number and prepared the solutions of medicines based on the number appeared on the paper slip, either #1 or #2. Then, the unlabeled syringes of solutions were handed over to an anesthesiologist who was administrating the medicines and performing general anesthesia. Patients, the anesthesiologist who performed anesthesia and another anesthesiologist who observed and recorded the data were all blinded to the medication patient had received. All anesthesia operations and monitoring were expected to be completed by two fixed anesthesiologists.

After arrival in the operating room, all patients were continuously monitored pulse oxygen saturation, heart rate, electrocardiogram, and non-invasive blood pressure (invasive arterial blood pressure monitoring if necessary). After pre-oxygenation via face mask, anesthesia was induced with intravenous 2 mg/kg propofol, 0.3 μg/kg sufentanil, 0.2 mg/kg cisatracurium and 1.5 mg/kg lidocaine (Sinopharm Group Rongsheng Pharmaceutical Co., Ltd., Henan Wuzhi, batch number: H20043676) over 10 s (lidocaine group) or same volume of normal saline (control group), followed by 2 mg·kg^−1^·h^−1^ continuous lidocaine (lidocaine group) or normal saline (control group) until the end of the surgery. Tracheal intubation was performed, and anesthesia was maintained with 0.1 μg·kg^−1^·min^−1^ remifentanil continuous intravenous infusion. By adjusting the concentration of sevoflurane, bispectral index was maintained between 40—60. If necessary, added cisatracurium 2 mg to ensure the muscle relaxation required for surgery. When the heart rate was greater than 100 beats/minute or the mean arterial pressure increases by 15%, added sufentanil 5 μg. When the operation completed, a mixture of 2 μg/kg sufentanil and 10 mg/kg lidocaine (lidocaine group)or 2 μg/kg sufentanil (control group) was continuously intravenous infused by postoperative patient-controlled intravenous analgesia pump (100 ml). An initial loading dose consisted of 5 ml of the pump. The background dosing rate was 2 ml/h, and lasted for 48 h. The patient-controlled dosing was 0.5 ml for every successful trigger with a lockout interval of 15 min.

At the end of surgery, the tracheal tube was removed and the patient was sent to the post anesthesia care unit (PACU). All patients had previously received guidance on intravenous PCA and numerical rating scales (NRS), ranging from grade 0 (no pain) to grade 10 (most severe pain). Stable vital signs and Numerical Rating Scale (NRS) < 4 were the criteria for discharge from the PACU. If NRS ≥ 4, 5 mg dezocine (a potent opioid analgesic like morphine) was used as a rescue medication.

Gender, age, body mass index, duration of operation (from the beginning of the skin incision to the end of the suture), and the use of anesthetics were recorded. The primary outcome of the study was the incidence of CPSP in the two groups of patients after surgery at 3 months. The secondary outcome was the incidence of CPSP in the two groups of patients after surgery at 6 months; the effect of perioperative intravenous infusion lidocaine on postoperative pain within the first 24 and 48 h; total amount of sufentanil (including intraoperative dosage and information automatically provided by the intravenous PCA pumps); the number of PCA triggers within 48 h after surgery. Patients were followed by a research assistant who did not know the treatment plan at 3 and 6 months after the operation. The research assistant by asking for medical history, physical examination, and questionnaire. After excluded other potential causes of pain, such as psychological factors, recurrence or metastasis of lung cancer, etc., asked whether the patient had experienced persistent pain or chronic onset due to surgery. If his/her answer was yes, then it was marked as suffering from CPSP. Further questions were as follows: Can you rate your pain from 0 to 10? Where was the painful part? How did the pain occur, caused by stretching, coughing or spontaneous? Was your pain intermittent or continuous? What medicine did you take to relieve the pain? If taken, was the medicine over the counter medicine or prescription drugs?

Our preliminary study showed that the incidence of CPSP at 3 months was 11% in lidocaine group and 44% in control group. Considering alpha of 0.05 and power of 80, and 10% shedding rate, so at least 56 cases were needed as the initial sample size. In order to increase the sample size as much as possible and avoid the loss of follow-up patients, 64 patients were finally included. Statistical analysis was performed using SPSS23 statistical software program (SPSS Inc., Chicago, IL). Shapiro–Wilk test was used to examine the normality of distribution of data. All continuous data were presented as the mean with standard deviation or as median. Categorical data are expressed as count and percentage (%). Continuous variables were compared using the Student t-test or Mann–Whitney U test if the data were not distributed normally. Categorical variables were compared using the chi-square or Fisher exact test, as appropriate. *p* < 0.05 was considered to be statistically significant.

## Results

**Fig. 1:**
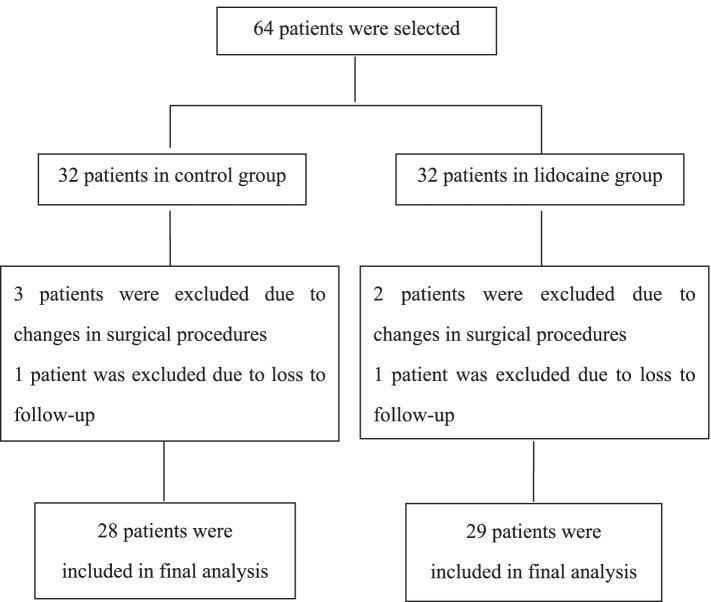
Flow diagram showing the procedure used in this study

From November 25, 2019 to June 28, 2021, a total of 64 patients were initially enrolled, 5 cases were excluded due to changes in surgical procedures and 2 cases were excluded due to loss to follow-up. Finally, a total of 57 patients, including 24 males and 33 females completed in this study, detailed in Fig. 1 There were no significant differences in age, body mass index (BMI), gender, American Society of Anesthesiologists (ASA) classification and operation duration between the two groups (all, *p* > 0.05; Table 1).

**Table 1:** The general characteristic data in the two groups

Variables	Control group (*n* = 28)	Lidocaine group (*n* = 29)	*p-*value^*^
Age (yrs)	54.25 ± 8.51	55.10 ± 8.08	0.70
BMI (kg/m^2^)	22.71 ± 2.53	22.56 ± 2.20	0.82
Gender (m/f)	12/16	12/17	0.91
Duration of operation (min)	95.00 ± 25.79	101.00 ± 34.96	0.47
ASA classification			0.21
II	10	6	
III	18	23	

Data are presented as mean (SD) or median (IQR) for continuous measures, and n (%) for categorical measures

*BMI* Body mass index, *ASA* American Society of Anesthesiologists

^*^ Independent Student t-test *p* value or Chi-Square *p* value as appropriate

Fifty-seven patients undergoing thoracoscopic radical pneumonectomy were followed up 3 and 6 months later. Compared with the control group, the incidence of CPSP 3 months in the lidocaine group was lower (13 patients, 46.4% vs. 6 patients, 20.7%, *p* = 0.04). There was no statistically significant difference in NRS score between the control group (2.61 ± 0.87) and the lidocaine group (2.66 ± 1.37) (*p* > 0.05), as shown in Table 2. The incidence of chronic postoperative pain 6 months after the surgery (4 patients, mild 3 cases, moderate 1 case, 14.3% vs. 4 patients, mild 4 cases, 13.8%) and its NRS score (2.00 ± 1.41 vs. 1.25 ± 0.50) were no significant differences in the lidocaine group (Table 3). The NRS pain scores at 24 h (1.68 ± 0.72 vs. 1.90 ± 0.86) and 48 h (1.21 ± 0.42 vs. 1.20 ± 0.41) after the operation were no significant difference in the lidocaine group. No one used rescue medication either in the PACU or the ward.

**Table 2:** Occurrence and characteristics of chronic postoperative pain in 3 months after operation

Variables	Control group (*n* = 28)	Lidocaine group (*n* = 29)	*p*-value	Total
Number of people with CPSP	13	6	0.04^*^	19
NRS	2.61 ± 0.87	2.66 ± 1.37	0.92	
Pain degree			0.56	
Mild	11	5		16 (84.2%)
Moderate	2	1		3 (15.8%)
Severe	0	0		0 (0.0%)
Location			0.78	
Partial incision	6	4		10 (52.7%)
Chest wall	7	2		9 (47.3%)
Pain form			0.38	
Spontaneous pain	3	3		6 (31.6%)
Stretch pain	8	3		11 (57.9%)
Cough pain	2	0		2 (10.5%)
Duration			0.32	
Always	0	1		1 (5.3%)
Intermittent	13	5		18 (94.7%)
Analgesic use			1.00	
Unused	11	5		16 (84.2%)
Used	2	1		3 (15.8%)

*CPSP* Chronic postsurgical pain, *NRS* Numerical rating scales

^*^Chi-square test, *P* < 0.05

**Table 3:** Chronic postoperative pain in 6 months after operation

Variables	Control group (*n* = 28)	Lidocaine group (*n* = 29)	*p*-value	Total
Number of people with CPSP	4	4	1.00^a^	8
NRS	2.00 ± 1.41	1.25 ± 0.50	0.37^b^	
Pain degree				
Mild	3	4		7 (87.5%)
Moderate	1	0		1 (12.5%)
Severe	0	0		0 (0.0%)
Location			0.23^a^	
Partial incision	1	2		3 (37.5%)
Chest wall	3	2		5 (62.5%)
Pain form			0.11^a^	
Spontaneous pain	1	0		1 (12.5%)
Stretch pain	2	2		4 (50.0%)
Cough pain	2	1		3 (37.5%)
Duration			0.46^a^	
Always	0	1		1 (12.5%)
Intermittent	4	3		7 (87.5%)
Analgesic use			1.00^a^	
Unused	4	4		8 (100%)
used	0	0		0 (0.00%)

CPSP chronic postsurgical pain, NRS numerical rating scales

^a^ Fisher exact test, ^b^ NStudent t-test

Compared with the control group, the amount of the anesthetics used in the perioperative period was no significant difference in the lidocaine group, except the cumulative dose of sufentanil was significantly lower in the lidocaine group (149.64 ± 18.20 *vs.* 139.47 ± 16.75, *p* = 0.03). And the number of PCA triggers (8.21 ± 4.371 *vs.* 5.83 ± 4.12, *p* = 0.04) was significantly greater in the control group within 48 h after surgery (Table 4).

**Table 4:** Comparison of perioperative anesthetic drug consumption

Variable	Control group (*n* = 28)	Lidocaine group (*n* = 29)	*p*-value
Cisatracurium (mg)	15.93 ± 3.13	16.22 ± 3.98	0.76
Propofol (mg)	152.32 ± 29.07	142.93 ± 23.81	0.19
Sevoflurane (ml)	44.70 ± 17.84	48.58 ± 23.11	0.47
Remifentanil (μg)	638.32 ± 249.19	668.32 ± 330.62	0.70
Sufentanil cumulative dosage (μg)	149.64 ± 18.20	139.47 ± 16.75	0.03^*^
The number of PCA triggers (times)	8.21 ± 4.37	5.83 ± 4.12	0.04^*^

*PCA* patient-controlled analgesia

^*^Student t-test, *p* < 0.05

## Discussion

This study was designed to determine whether perioperative intravenous infusion of lidocaine has beneficial effects on chronic postsurgical pain in patients undergoing thoracoscopic radical radical pneumonectomy. As results, the incidence of chronic pain 3 months after the surgery was lower. That is consistent with reports in the literature [7, 8]. And also, a significant reduction in the cumulative dosage of sufentanil in perioperative period and the number of PCA triggers within 48 h after surgery were observed in the lidocaine group.

In terms of analgesia, one study [15] has reported that the patients received patient-controlled dose of 2 mg/kg lidocaine and after, continuous infusion of 3 mg·kg^−1^·h^−1^ until the tracheal tube was removed, failed to detect reductions in acute postsurgical pain scores and opioid consumption in the first 48 h in robotic thyroidectomy. However, reduced the incidence of 3 months CPSP in patients. In addition, the outcomes from a previous study carried out systemic lidocaine reduced the incidence and severity of 3 months CPSP after breast cancer surgery [16]. A 129 cases retrospective review of propofol-opioid usage in spine surgery indicated that a lidocaine infusion could be effectively reduced propofol and sufentanil usage without a negative effect [17]. Another study showed that continuous infusion of lidocaine (1.5 mg/kg later 2 mg·kg^−1^·h^−1^) resulted in reduction of sufentanil requirements during pediatric colonoscopy [18]. These findings were consistent with our research. All about those CPSP questions that patients were asked at follow-up have no significant difference in this study. Three patients who took medicine all chose over the counter medicine to relieve the pain. The difference was that we had a longer follow-up, suggesting that the pain at six months was not statistically significant.

The analgesic and anti-hyperalgesic properties of systemic lidocaine have been understood and recognised for almost 70 years [19]. It was initially thought that the canonical analgesic mechanism of lidocaine was to block the expression of voltage-gated sodium channels (VGSCs) in the dorsal root ganglia of injured axons and the terminal injury site (neuromas) [20]. However, ongoing research has showed that lidocaine may exert effects on various of other molecular targets (such as potassium channels, calcium channels, transient receptor potential channels, G-protein-coupled receptors, acetylcholine receptors, glutamate receptors, serotonin receptors and opioid receptors et al.) involved in acute and chronic pain [19]. Besides, lidocaine has anti-inflammatory properties. Sensitivity following tissue inflammation may be secondary to the release of chemical mediators, cytokines, and growth factors from inflamed tissue that bind to receptors on pain receptors [21]. Lidocaine can affect inflammatory cells and reduce the release of mediators of inflammation, such as IL-4, IL-6, and tumour necrosis factor-alpha [22]. These include effects on the central nervous system and peripheral nervous system, where lidocaine acts via modulation of inhibitory and excitatory neurotransmission, silencing ectopic discharges and suppression of inflammatory processes [19]. In addition, in clinical work, the duration of action of lidocaine usually significantly exceeds its plasma half-life. This is not only a complex correlation of multiple pathways, but may also be associated with synergistic effects of other pain medications, such as opioids [19].

Intravenous infusion of lidocaine during the perioperative period has been increasingly used in clinical practice, while the accumulation of drugs for continuous infusion of lidocaine is a problem worthy of attention. The dosage regimen in most studies used a loading dose of 1–2 mg/kg and an intraoperative maintenance dose of 2–4 mg·kg^−1^·h^−1^, with a blood concentration of 1–3 μg/ml [23]. In the currently published clinical literature on intravenous infusion of lidocaine, even after continuous infusion of lidocaine at a rate of 1.33 mg·kg^−1^·h^−1^ for 24 h, the level of plasma drug concentration was still far below the toxic level (5 μg/ml) [23]. There were no reports of major adverse events related to intravenous lidocaine infusion. In the present trial, none of the patients experienced lidocaine-related adverse reactions.

There were some limitations associated with this study. Firstly, the sample size was limited. Increasing the sample size was beneficial to reduce sampling error. Secondly, we did not monitor the plasma concentration of lidocaine. Plasma concentration of lidocaine might be unstable during the perioperative period, especially during postoperative PCA, which might impair its analgesic efficacy. However, lidocaine wakened spontaneous pain, allodynia, or hyperalgesia within a certain range of plasma concentrations, and the duration of action exceeded plasma half-life. Besides that, the mechanism of intravenous infusion of lidocaine to relieve postoperative pain has not been clear. Continuously measured cytokines and inflammatory factors at multiple points during perioperative period is beneficial to understand the analgesic mechanism of lidocaine.

## Conclusions

In conclusion, perioperative infusion lidocaine significantly reduced the number of PCA triggers, and the incidence of chronic postoperative pain 3 months after the surgery, which provides another possible way for the management of postoperative acute and chronic pain.

## Data Availability

The datasets used and/or analysed during the current study available from the corresponding author on reasonable request.

## Abbreviations

CPSP: Chronic postsurgical pain
BMI: Body mass index
ASA: American Society of Anesthesiologists
PACU: PPost anesthesia care unit
NRS: Numerical rating scales
PCA: Patient-controlled intravenous analgesia
